# Public health impact and cost-effectiveness of rotavirus vaccination in China: Comparison between private market provision and national immunization programs

**DOI:** 10.1080/21645515.2022.2090162

**Published:** 2022-07-11

**Authors:** Jiahao Wang, Haijun Zhang, Haonan Zhang, Hai Fang

**Affiliations:** a Department of Health Policy and Management, School of Public Health, Peking University, Beijing, China; b China Center for Health Development Studies, Peking University, Beijing, China; c School of Health Humanities, Peking University, Beijing, China; d Peking University Health Science Center-Chinese Center for Disease Control and Prevention Joint Center for Vaccine Economics, Beijing, China; e Key Laboratory of Reproductive Health National Health Commission of the People’s Republic of China, Beijing, China

**Keywords:** Diarrhea, rotavirus vaccine, economic evaluation, cost-effectiveness, vaccination, China

## Abstract

In China, progress to include the RV vaccine in the national immunization program (NIP) is slow. The only two vaccines, the Lanzhou lamb rotavirus vaccine (LLR) and Rotateq, are provided through the private market. This study aims to assess the health impact and cost-effectiveness of using three vaccines in the NIP, Rotateq, Rotarix, and LLR, compared to the status quo. A decision-tree Markov model was adopted to follow the 2019 birth cohort, and a societal perspective was used. Input parameters were based on the latest local data when possible. Outcomes included cases and deaths averted, quality-adjusted life years (QALYs) gained, and incremental cost-effectiveness ratios (ICER). Sensitivity analyses and scenario analyses to consider herd immunity and vaccine price reduction were performed. Including Rotateq in the NIP was projected to prevent 348 million RVGE cases (62.6% reduction) and 4251 deaths (72.6% reduction) compared to the status quo. Rotarix through the NIP would prevent 48.7% of cases and 63.2% of deaths, and LLR would avert 20.3% of cases and 22.4% of deaths. The ICERs per QALY gained were US$ 8833 for Rotateq through the NIP, US$ 9503 for Rotarix, and US$ 26,759 for LLR. In uncertainty analyses, the reduction of vaccine prices and the incorporation of herd immunity further improved the cost-effectiveness of the NIPs, especially Rotateq or Rotarix. In conclusion, introducing the RV vaccine in China’s NIP is expected to be cost-effective compared to the GDP per capita. Reducing vaccine prices and adopting vaccines with better efficacy would be the future focus.

## Introduction

Rotavirus (RV) is the leading cause of severe acute gastroenteritis (GE) in children under five years of age (U5) worldwide and causes approximately 258 million cases, 2 million hospitalizations, and 128 thousand deaths annually.^1–7^ As the improvements in water, sanitation, and hygiene haven’t substantially altered the incidence of RV disease, vaccination is considered one of the most efficient interventions to reduce morbidity and mortality from RV.^1,2,7,8^ Since 2009, the World Health Organization (WHO) has recommended all countries include RV vaccines in their national immunization programs (NIPs).^2,9^ Four RV vaccines (RotaTeq, Rotarix, Rotavac and ROTASIIL) have been prequalified by WHO; among them, Rotarix (RV1) and Rotateq (RV5) have been widely used internationally.^1,2^ As of January 2022, 114 countries have introduced rotavirus vaccines, and significant reductions in disease burden and effectiveness of RV vaccines has been observed.^10–13^

China has a substantial disease and economic burden of RVGE, with an estimated 12 million cases in 2008 and 3200 deaths in 2013 among U5 children.^14,15^ China has the highest societal treatment costs for RVGE in Asia.^16^ However, progress in immunization planning is relatively slow compared to other countries.^17,18^ Currently, only two RV vaccines are available in China: Lanzhou lamb rotavirus vaccine (LLR) by a domestic manufacturer, Lanzhou Institute of Biological Products (licensed in 2000) and RotaTeq (newly licensed in September 2018).^17,18^ Moreover, under the immunization financing mechanism in China, RV vaccines are classified as non-Expanded Program on Immunization (EPI) vaccines, and are provided through the private market at a high out-of-pocket prices ($26- 43/dose), resulting in a low vaccine coverage across the country and potential disparities in vaccination access and health outcomes.^19,20^ Without vaccination or timely treatment, RVGE could cause severe fluid and electrolyte imbalance and the development of fatal complications, leading to shock and even death.^1,21,22^ Lack of RV vaccine in the NIP might contribute to disease burden and prolonged hospitalization, health care expenses, and social productivity loss.^1,21,22^ To date, few studies in China have assessed the cost-effectiveness of including RV vaccine in the NIPs, and results are limited due to both the uncertainty in local parameters (vaccine efficacy, price, etc.) at the time of the study and the assumption of zero coverage in the status quo, which may overestimate the cost-effectiveness of the strategies of NIPs.^23–26^

Given the availability of relevant evidence recently, particularly on the epidemiology of RVGE, vaccine profile, and coverage in the private market provision in China, and updates to economic evaluation models internationally, this study aims to evaluate the public health impact and cost-effectiveness of introducing rotavirus vaccine into the NIPs compared to the current provision by private market in China. By integrating the latest local data, if available, the results would contribute to evidence-based decision-making in China’s NIPs, as well as to the prevention and reduction of regional RVGE disease and economic burden.

## Materials and methods

### Study design and model structure

**Figure 1.**
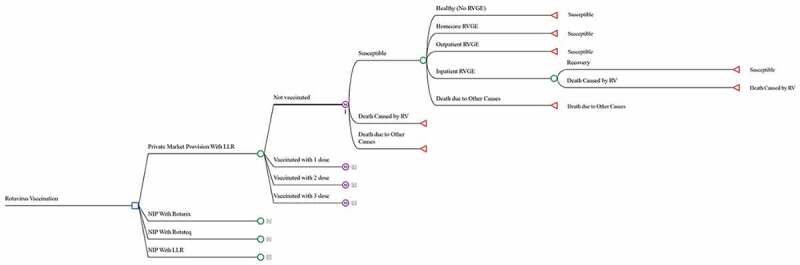
Overview of the decision tree-Markov model.

An economic evaluation was conducted to compare the impact and cost-effectiveness of the potential national rotavirus vaccination programs with the status quo, private market provision. The two RV vaccines prequalified by WHO, RotaTeq and Rotarix, and the domestic LLR were evaluated.^9,18^ LLR and Rotateq have been licensed in China, and Rotarix has been used internationally with a randomized controlled trial (RCT) demonstrating its efficacy among Chinese children.^18,27^ The study didn’t include the other two WHO prequalified vaccines, Rotavac and ROTASIIL, as they were predominantly used locally in India and were not available in the Chinese market.^9,18^ A decision tree-Markov state transition model was developed to estimate the impact of rotavirus vaccine on disease burden, quality-adjusted life years (QALYs), and costs for the 2019 birth cohort in China (Figure 1). The model follows the 2019 Chinese birth cohort for 5 years with a cycle length of 6 months, and allows children to have more than once RVGE episode during the horizon. For each cycle, children will either stay healthy or develop an RVGE episode. Children with RVGE may require home-based care or seek outpatient or inpatient medical care. It is assumed that RVGE requiring home care or outpatient visits is not severe (Vesikari severity score <11), and that only hospitalized cases are severe (severity score ≥11) and may result in death. Children would develop partial immunity against subsequent RVGE when they have been vaccinated against rotavirus or when they are exposed to a natural rotavirus infection for the first time.^1,7,28^

The analysis was conducted from a societal perspective using a lifetime time horizon for the cohort. All costs and impacts were discounted at 3% following WHO’s recommendations, and all costs were converted to 2019 US dollars (1 US$ = 6.5 RMB) with adjustment for inflation where necessary.^29,30^ The model was developed using TreeAge Pro 2019 (TreeAge Software, Inc., Williamstown, MA). The input parameters for the model are presented in Table 1.

**Table 1.** Model parameters and data source.

Parameters	Base case	Range	Distribution	Source
**Demographic parameters**				
2019 birth cohort (thousand)	14650	N/A	Not varied	^72^
Neonatal mortality rate (per 100,000 children)	375	334-419	Beta	^32^
Infant mortality rate (per 100,000 children)	593	547-643	Beta	^32^
Child mortality rate age 1–4 (per 100,000 children)	199	170-209	Beta	^32^
Under-five mortality rate (per 100,000 children)	791	730-857	Beta	^32^
**Epidemiology parameters**				
Annual incidence of RVGE (per 100,000 children under 5)	8933	6829–11488	Beta	^31,34^
Annual death rate of RVGE (per 100,000 children under 5)	9.7	9.0–10.5	Beta	^32,33,75^
Age distribution of RVGE				^36^
0-5m	12.3%	N/A	Not varied	
6-11m	30.3%	N/A	Not varied	
12-23m	39.9%	N/A	Not varied	
24-35m	10.4%	N/A	Not varied	
36-47m	5.4%	N/A	Not varied	
48-59m	1.7%	N/A	Not varied	
Care-seeking rate	74.8%	56.1–93.5%	Beta	^37–40^
Ratios of the number of outpatient visits to hospital admissions	10	5 -15	Triangular	^35^
Duration of RVGE (days)				^15^
Home care or outpatient case	3	1-5	Triangular	
Hospitalization	6	2-11	Triangular	
**Vaccine characteristics and natural acquired immunity**				
Vaccine efficacy				
Rotateq against homecare or outpatient visit, 3 doses	69.3%	54.5–79.7%	Beta	^51^
Rotateq against hospitalization, 3 doses	78.9%	59.1–90.1%	Beta	^51^
Rotarix against homecare or outpatient visit, 2 doses	58.1%	44.3–68.8%	Beta	^37^
Rotarix against hospitalization, 2 doses	72.0%	54.1–83.6%	Beta	^37^
LLR against homecare or outpatient visit, 1 dose	34.9%	5.3–55.3%	Beta	^55^
LLR against hospitalization, 1 dose	39.0%	5.0–61.0%	Beta	^54^
Ratios of efficacy of 1 dose to 2 doses Rotarix	88.0%	82.0–100.0%	Triangular	^52^
Ratios of efficacy of 1 dose to 3 doses Rotateq	80.0%	62.0–83.0%	Triangular	^52^
Ratios of efficacy of 2 doses to 3 doses Rotateq	97.0%	90.0–100.0%	Triangular	^52^
Protection from the natural infection				
Efficacy against subsequent homecare or outpatient visit	73.0%	50.0–86.0%	Beta	^28^
Efficacy against subsequent hospitalization	87.0%	55.0–96.0%	Beta	^28^
Vaccine coverage in the current private market				^49^
1 dose LLR	20.26%	18.23–22.23%	Triangular	
2 doses LLR	5.51%	4.96–6.06%	Triangular	
3 doses LLR	1.77%	1.60–1.95%	Triangular	
Vaccine coverage in national immunization program				^50^
DTP4	91.05%	81.95–91.05%	Triangular	
DTP3	8.39%	7.55–8.39%	Triangular	
DTP2	0.13%	0–0.13%	Triangular	
DTP1	0.09%	0–0.09%	Triangular	
**Costs of treatment and immunization (US$)**				
Cost per home care case				^23,59^
Direct medical cost	3.5	2.6–4.4	Gamma	
Direct nonmedical cost	0.5	0.4–0.6	Gamma	
Indirect cost	6.8	5.1–8.5	Gamma	
Cost per outpatient case				^35^
Direct medical cost	67	50.2–83.7	Gamma	
Direct nonmedical cost	21.7	16.3–27.1	Gamma	
Indirect cost	78.6	59.0–98.3	Gamma	
Cost per inpatient case				^35^
Direct medical cost	236.2	177.1–295.2	Gamma	
Direct nonmedical cost	45.7	34.3–57.2	Gamma	
Indirect cost	148	111–185.1	Gamma	
Discounted lifetime productivity per capita	118470.3	88852.7–148087.8	Gamma	^62,63^
Vaccine price per dose				
Rotateq	43.1	38.8–47.4	Gamma	^64^
Rotarix	62.3	56.1–68.5	Gamma	Assumption
LLR	26.5	23.8–29.2	Gamma	^64^
Cost of immunization delivery per dose	10.4	7.8–13.0	Gamma	^66^
Vaccine wastage rate	0.05	0–10%	Beta	Assumption
**Utilities**				^45^
home care RVGE	0.685	0.582–0.788	Beta	
Outpatient RVGE	0.660	0.561–0.759	Beta	
Inpatient RVGE	0.591	0.502–0.680	Beta	
**Discount rate**	3%	0–10%	Uniform	Assumption

### Model input parameters

#### Disease burden

The disease burden of RVGE was estimated based on the Global Burden of Disease (GBD 2019), the UN Interagency Group for Child Mortality Estimation (UN-IGME), the China National Maternal and Child Health Surveillance System (MCHS), systematic reviews and population-based surveillance in China.^31–35^ The annual incidence of RVGE among U5 children was calculated based on the incidence of all-cause diarrhea in 2019 estimated by the GBD and the community detection rate of rotavirus in diarrhea (9.3%) in China.^31,34^ Using the method by the WHO Child Health Epidemiology Reference Group, the annual mortality rate of RVGE in 2019 was calculated by multiplying the under-five mortality rate by the proportion of deaths due to diarrhea (3.1%) from the MCHS and the RV detection rate in hospitalized diarrhea among U5 children (39.7%) from a systematic review.^5,6^ The age distribution of RVGE was obtained from population-based surveillance in northern and southern China.^36^ To estimate the number of cases by settings, cases were first differentiated as care-seeking and not care-seeking. Those not seeking care were categorized as home care cases. Considering the severity of diarrhea caused by RV, the care-seeking rate for RV diarrhea (74.8%) was assumed as the highest rate among those for diarrhea from Hospital Utilization and Attitudes Surveys (HUAS).^37–40^ Then, among those seeking cases, the ratio of the number of outpatient visits to hospitalization (149 vs. 14.4) from the surveillance in Beijing and Gansu provinces was applied to categorize outpatient and inpatient cases, adopting the methodology by Parashar et.al.^5,24,35^

Very few studies have examined the utility of children with RV diarrhea, and most of them were conducted in developed countries, such as the UK, Canada, and Danish.^41–45^ A study in Thailand reported the most updated results and was also the only one from developing countries.^45^ Considering the similarity in rotavirus epidemiology, healthcare system, and health service utilization of diarrhea between China and Thailand, the utility values were derived from the study in Thailand to estimate QALYs.^35,46,47^ The utility score for mild diarrhea was used for the homecare RVGE, and the score for moderate RVGE was used for outpatient RVGE and severe RVGE for inpatient RVGE. The average duration of RVGE and RVGE and its range for outpatients and inpatients were extracted from a hospital-based surveillance of rotavirus diseases conducted in 5 cities, including Beijing, Shanghai, Suzhou, Fuzhou, and Guangzhou, and the duration of homecare cases was assumed to be the same as that of outpatient cases.^15^

#### Vaccine characteristics and natural immunity

For the three-dose Rotateq and two-dose Rotarix, the vaccination was assumed to be delivered in conjunction with the Diphtheria, Tetanus, and Pertussis (DTP) vaccine according to the schedule recommended by WHO.^2,9^ The three-dose LLR was assumed to be administered according to the schedule recommended by the manufacturer: one dose in the first year followed by annual booster dose in the second and third years.^48^ By 2019, LLR was the most used vaccine in the private market, while RotaTeq was newly licensed in September 2018 with little availability and coverage information.^18,20,49^ It was assumed that the coverage for Rotateq was 0% in the status quo. The coverage for each dose of LLR vaccine in the status quo were estimated from a facility-based survey in 2019, covering over 6,000 children in 10 provinces.^49^ For RV vaccine coverage in the NIP using the three vaccines, respectively, the DTP vaccine coverage in China was used as a proxy.^50^

The efficacy of Rotateq and Rotarix was obtained from randomized controlled trials (RCT) conducted in China.^27,51^ Based on the meta-regression of global RCTs, where efficacy was estimated by duration of follow-up, the partial-immunization efficacy of vaccines was estimated by obtaining the ratios of efficacy between incomplete and complete courses of vaccination in a low-mortality stratum setting to which China belongs.^52,53^ Data from RCTs on the efficacy and protection duration of LLR were lacking, but case-control studies reported the effectiveness of 1 dose of LLR in the first two years.^54,55^ The efficacy of LLR was estimated from the studies, and it was assumed that 1 dose provided protection for 2 years, 2 doses for 4 years, and 3 doses for the whole course of 5 years.^54,55^ Birth cohort studies have demonstrated the protection from natural infection for subsequent infections, and we estimated the natural acquired immunity against subsequent infections based on the study in Mexico, which is also in the low-mortality stratum setting as China.^28,56,57^ The efficacy of vaccination and naturally acquired immunity against RVGE that required different types of care was distinguished based on the efficacy against RVGE of different severity. The efficacy against non-severe (Vesikari score <11) was used as the proxy for the efficacy against home care and outpatient RVGE, and the efficacy against severe (Vesikari score ≥11) was used as the proxy for the efficacy against inpatient RVGE. Adverse effects of vaccination were not incorporated based on the Cochrane review, clinical trials, and surveillance of adverse events following immunization in China.^8,27,51,54,55,58^ The impact of the herd immunity was incorporated in the uncertainty analyses.

#### Costs of health care and immunization

The health care costs for each RVGE included direct medical, direct non-medical, and indirect costs. Direct non-medical costs covered items such as transportation, accommodation and nonprescription medication. Indirect costs were associated with the loss of caregiver productivity. The costs of homecare case were estimated from the Hospital Utilization and Attitudes Surveys (HUAS) on diarrhea diseases in Zhejiang provinces, which covered 4400 children in areas of different socio-economic levels.^23,59^ The costs for inpatient and outpatient cases were obtained from the hospital-based active surveillance of RVGE among U5 children in Beijing and Gansu province.^35^ The direct medical cost were adjusted using health care expenditures by region from the China Health Statistical Yearbook, and direct non-medical costs were adjusted using the consumer price index (CPI) from the China Statistical Yearbook.^60,61^ Using the human capital approach, lifetime productivity loss due to premature death was estimated using the national average annual wage rate for the average years of productive life (16 to 60 years) from census data and discounted to the year of vaccination.^62,63^

The price of LLR and Rotateq in the private market were obtained from the Chinese Center for Disease Control and Prevention (CDC).^64^ The price of Rotarix, which has not yet approved in China, was estimated by multiplying the price of Rotateq by the ratio between the prices of Rotateq and Rotarix in the US market.^65^ The costs of immunization delivery per dose, including the direct costs for governments to provide immunization services (e.g, personnel, cold chain, surveillance, communication activities, training, and supervision) and the indirect costs for households to seek vaccination (e.g., transportation and loss of caregiver productivity), were derived from the cost and investment survey of routine immunization services in 15 provinces by the Chinese CDC.^62,66^ The cost of routine immunization service per dose was used as the proxy for the three RV vaccines’ immunization delivery costs per dose.

### Cost-effectiveness analysis

The cost-effectiveness results were presented as incremental cost-effectiveness ratios (ICERs), defined as the incremental costs, i.e., the cost of RV vaccination and the cost of disease, per RVGE case averted, per death averted and per QALY gained by the potential NIP strategies compared to the status quo. China has no policy or recommendation of thresholds for interpreting cost-effectiveness results, while one time and three-time the national gross domestic product (GDP) per capita were widely adopted.^23,26,67,68^ To reflect the conventional practice in China and the updated international opinion on the cost–effectiveness criteria, two thresholds were used for the evaluation: (1) the national GDP per capita in 2019 (US$ 10,276); (2) the threshold range proposed by Woods et al. for China (US$ 1151–4550), which accounted for the opportunity cost of the health expenditure and may be more appropriate to inform on resource allocation decisions.^68–72^

### Uncertainty analyses

Uncertainty analyses, including sensitivity analyses and scenario analyses, were performed to test the robustness of the model results and to assess the sources of model uncertainty. Deterministic sensitivity analyses (DSA) were conducted for all model parameters using the plausibility ranges specified in Table 1. For parameters with an unknown uncertainty range, the plausibility range was assumed to be 25% of the base value. Probabilistic sensitivity analysis (PSA) was conducted using Monte Carlo simulation (N = 1000 iterations) to assess the impact of changing multiple parameters simultaneously. The model uncertainty from the DSA and PSA were summarized using tornado diagrams and cost-effectiveness acceptability curves.

In scenario sensitivity analyses, the vaccine price per dose in the NIPs for the three RV vaccines was adjusted, and the impact of herd immunity was considered. The combined effect of the vaccination (direct and herd immunity effects) at different levels of RV vaccine coverage were estimated using regression estimates proposed by Wahl et al.^62,73^ The regression model was detailed in supplementary material 1. The price of RV vaccines would be reduced in the private market by 10%, 25%, and 50% to estimate the impact of the potential vaccine prices in the NIPs on cost-effectiveness outcomes, and identified the break-even price at which the incremental vaccination costs in the NIPs would be offset by the averted economic burden of the disease compared to the status quo. In addition, a threshold analysis was conducted to explore the price at which Rotarix would be as cost-effective as Rotateq at the current market price.

## Results

### Health benefits and economic impacts of rotavirus vaccination

Table 2 shows the health benefits, economic impact, and ICERs of the three national rotavirus vaccination programs compared with the current provision by the private market. The status quo was projected to cause 5.57 million RVGE cases, including 1.41 million homecare cases, 3.80 million outpatient cases and 366 thousand inpatient cases, and 5855 deaths among the 2019 birth cohort over five years. In comparison, national rotavirus vaccination programs could substantially reduce the disease burden. Including Rotateq in the NIP would cause the disease burden of 2.09 million RVGE cases and 1604 deaths, reducing 62.6% of the total cases and 72.6% of the deaths and gaining additional 131,850 QALYs from the status quo. Rotarix through the NIP would cause the disease burden of 2.86 million RVGE cases and 2157 deaths, averting 48.7% of RVGE cases and 63.2% of the deaths and gaining additional 113,913 QALYs from the status quo. LLR through the NIP would avert 20.3% of RVGE cases and 22.4% of deaths with an additional 41,185 QALYs gained.

**Table 2.** Disease burden averted and cost-effectiveness of including RV vaccines into NIP compared with the status quo.

Items	Status quo: Private Market Provision	NIP: Rotateq		NIP: Rotarix		NIP: LLR	
	Results	Results	Difference	Results	Difference	Results	Difference
**Disease burden**							
Total cases	5,572,196	2,085,635	−3,486,561	2,860,061	−2,712,135	4,442,339	−1,129,857
Home care cases	1,407,505	536,766	−870,739	736,790	−670,715	1,124,263	−283,242
Outpatient cases	3,798,030	1,448,416	−2,349,614	1,988,163	−1,809,867	3,033,726	−764,304
Inpatient cases	366,661	100,453	−266,208	135,108	−231,552	284,350	−82,311
Total Death	5,855	1,604	−4,251	2,157	−3,698	4,541	−1,314
**Cost** (million US$)							
Total costs	1,659.85	2,824.54	1,164.69	2,742.38	1,082.53	2,761.93	1,102.08
Cost of treatments	808.24	291.30	−516.94	398.66	−409.58	641.93	−166.31
Cost of premature deaths	693.65	190.04	−503.61	255.60	−438.05	537.93	−155.72
Immunization costs	157.96	2,343.20	2,185.24	2,088.12	1,930.16	1,582.07	1,424.11
**Effectiveness**							
QALYs gained	438,988,784	439,120,634	131,850	439,102,697	113,913	439,029,969	41,185
**ICERs**							
US$ per case averted	**/**	334	/	399	/	975	/
US$ per death averted	**/**	273,984	/	292,770	/	838,475	/
US$ per QALY gained	/	8,833	/	9,503	/	26,759	/

From a societal perspective, the total incremental cost of introducing Rotateq into the NIPs is projected to be 1164.69 million US$ compared to the status quo, with the additional cost of immunization (2185.24 million US$) being partially offset by the averted cost of treatments and the lost productivity from premature death (1020.55 million US$). The introduction of Rotarix into the NIPs could generate an incremental cost of 1082.53 million US$, while including LLR in the NIP with LLR could generate an incremental cost of 1102.08 million US$.

### Cost-effectiveness analysis

Compared to the status quo, the ICERs of Rotateq through the NIP were US$ 334 per case averted, US$ 273,984 per death averted, and US$ 8833 per QALY gained. The ICER per QALY gained was US$ 9503 for Rotarix and US$ 26,759 for LLR. At a threshold of one-time GDP per capita (US$ 10,276), Rotateq and Rotarix through the NIP were cost-effective. But all the three RV vaccines were not cost-effective at the threshold range advised by Woods et al. (US$ 1151–4550) (Table 2).

### Uncertainty analyses

**Figure 2.**
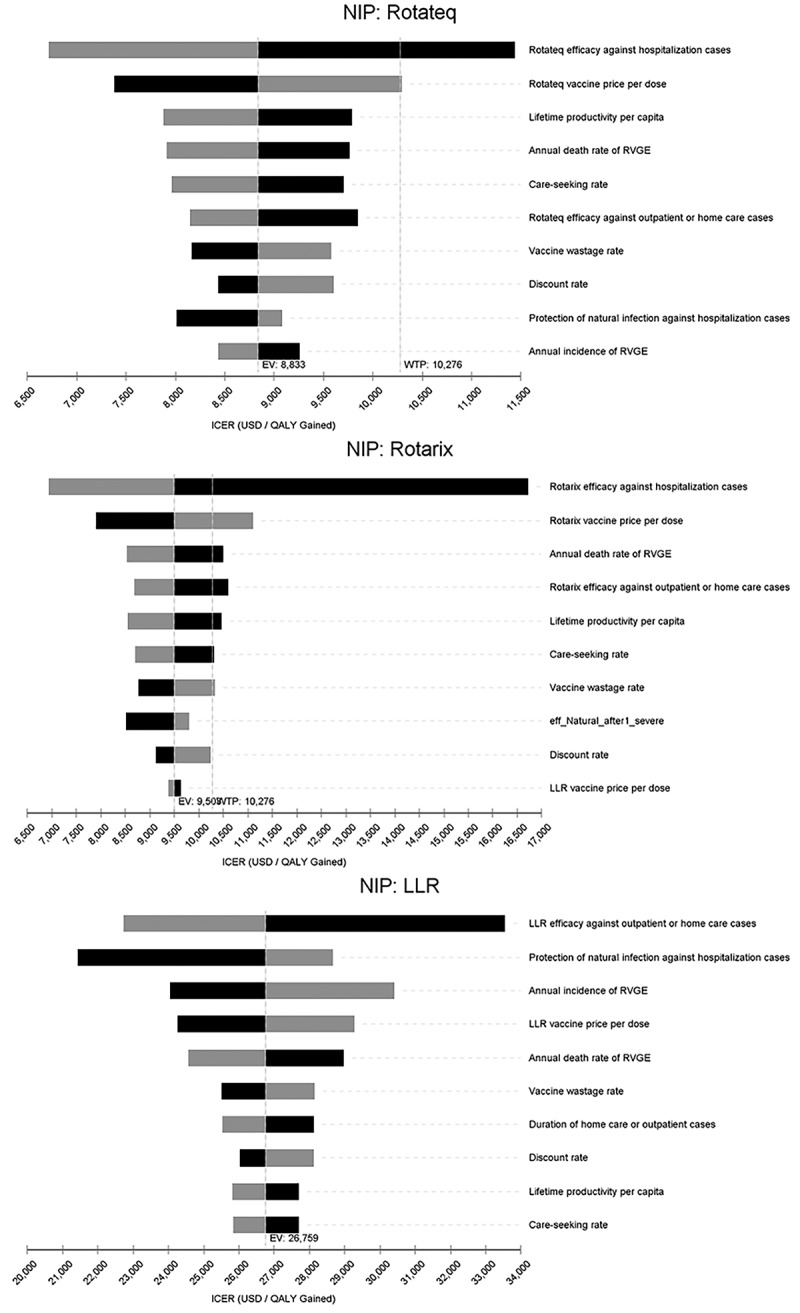
Tornado diagrams for one-way sensitivity analyses of NIP with Rotateq, Rotarix or LLR compared to the status quo.

**Figure 3.**
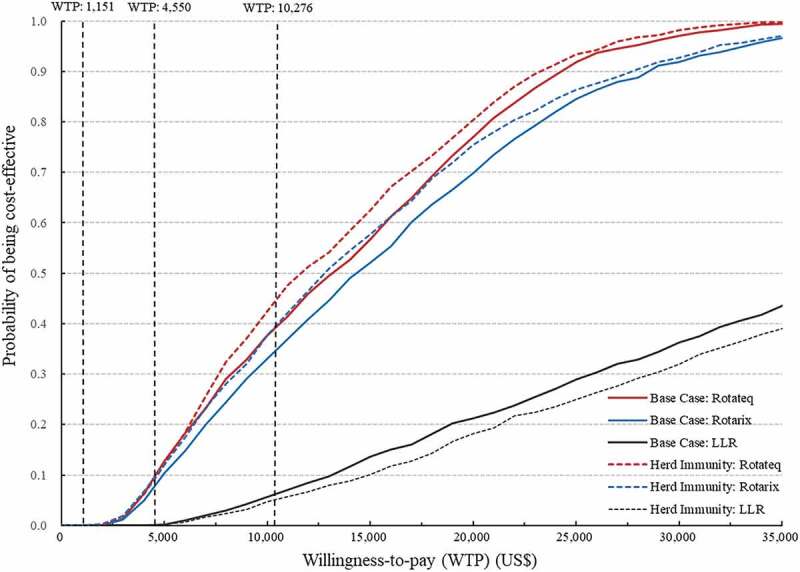
Cost-Effectiveness acceptability curves of the PSAs for the NIPs with Rotateq, Rotarix or LLR in the base case scenario and the scenario of considering herd immunity.

In the deterministic sensitivity analysis, the most sensitive parameters for Rotateq or Rotarix through the NIP were vaccine efficacy against hospitalization and vaccine price. The most sensitive parameters for LLR were vaccine efficacy against outpatient or home care cases and protection of natural infection against hospitalization (Figure 2). In the PSA, the probability of being cost-effective at a threshold of one-time GDP per capita was 38.7% for Rotateq, 34.3% for Rotarix, and 6.3% for LLR through the NIP. The probability of being cost-effective at the upper bound (US$ 4550) of the threshold range by Woods et al. was 9.3% for Rotateq, 7.9% for Rotarix, and 0.1% for LLR through the NIP (Figure 3).

The scenario analysis showed that including the herd immunity in the model reduced the ICER of Rotateq to US$ 8434 per QALY, Rotarix to US$ 9097 per QALY, but slightly increased the ICER of LLR to US$ 29,210 per QALY (Table 3). In the PSA, after considering the herd immunity, the probability of being cost-effective at a one-time GDP per capita threshold increased to 44.0% for Rotateq and 38.5% for Rotarix, but decreased to 5.3% for LLR. The probability of being cost-effective at the upper bound (US$ 4550) of the threshold range by Woods et al. increased to 9.5% for Rotateq and 9.6% for Rotarix (Figure 3).

**Table 3.** Scenario analyses of the NIPs with Rotateq, Rotarix or LLR at different vaccine prices in the base case scenarios and the scenario of considering herd immunity.

Vaccine price adjustment	NIP: Rotateq			NIP: Rotarix			NIP: LLR		
	Price (US$/dose)	Base case (US$/QALY gained)	Herd immunity scenario (US$/QALY gained)	Price (US$/dose)	Base case (US$/QALY gained)	Herd immunity scenario (US$/QALY gained)	Price (US$/dose)	Base case (US$/QALY gained)	Herd immunity scenario (US$/QALY gained)
Base	43.1	8,833	8,434	62.3	9,503	9,097	26.5	26,759	29,210
10% reduction	38.8	7,377	7,014	56.1	7,903	7,537	23.9	23,891	26,139
25% reduction	32.3	5,192	4,883	46.7	5,502	5,197	19.9	19,591	21,532
50% reduction	21.6	1,550	1,331	31.2	1,500	1,297	13.3	12,423	13,854
Break-even^a^	17.5	181	/	26.0	177	/	1.3	Cost-saving	/
Break-even^b^	17.0	/	Cost-saving	25.3	/	Cost-saving	1.8	/	584

^a^Herd Immunity Scenario.

^b^Base case scenario (without herd immunity).

In addition, the scenario analysis explored the ICERs of the RV vaccines through the NIP at varied vaccine prices in the absence and presence of herd immunity in the model. In the base case without herd immunity, the break-even price for Rotateq is US$ 17.0 per dose, a reduction of 60.6% from the current price. The break-even price is US$ 25.3 per dose for Rotarix (a 59.4% reduction) and US$ 1.8 for LLR (a 93.2% reduction). When herd immunity is included in the model, the break-even price is US$ 17.5 per dose for Rotateq (a 59.4% reduction), US$ 26.0 per dose for Rotarix (a 58.3% reduction) and 1.3 US$ for LLR (a 95.1% reduction). Moreover, the threshold analysis showed that Rotarix at 59.7 US$ per dose (a 4.2% reduction) would be as cost-effective as Rotateq with the current market price in both the base case scenario and the scenario that included the herd immunity.

## Discussion

To reduce the substantial RV disease burden, countries around the world, particularly in the western Pacific and southeast Asian regions, have advanced progress in evaluating rotavirus vaccines and introducing them into the NIPs.^10,12^ In China, the detection rate of rotavirus was 22.9% among outpatient diarrhea, and 39.7% among inpatient diarrhea.^74,75^ It was reported that the current prevalence characteristic of RV in China is similar to that of other countries before the introduction of RV vaccines.^74^ This study presents the comprehensive cost-effectiveness of using three potential RV vaccines in the NIPs, integrating the latest local data available in China and accounting for the impact of private market provision and herd immunity. The inclusion of RV vaccines in the NIP could substantially reduce the disease burden of RV diarrhea in children under 5 years of age in China compared to the current situation that results in 5.57 million RVGE cases and 5855 deaths in the birth cohort. For example, Rotateq through the NIP could avert 62.6% of the total cases and 72.6% of the deaths, with the ICERs of US$ 8833 per QALY gained. The national vaccination program, especially using Rotateq or Rotarix showed to be cost-effective compared with the national GDP per capita. Moreover, the reduction of vaccine prices and the incorporation of herd immunity have further improved the cost-effectiveness, especially with Rotateq or Rotarix.

The model parameter, data sources, and assumptions may have a significant impact on the results of the economic evaluation. To estimate the disease burden, we have collected the most updated, representative, and local data from multiple sources. The all-cause mortality rate, the proportion of deaths due to diarrhea, and RV detection rate among community and hospitalized diarrhea were obtained from UN-IGME, MCHS, and the systematic review and meta-analysis in China, respectively, to promote national representativeness.^32–34–75^ In the absence of national surveillance, we obtained the incidence of all-cause diarrhea in 2019 from the GBD.^31^ Based on global data from the scientific literature, population-representative surveys, and health care use records, the GBD adopted a Bayesian, hierarchical meta-regression tool as well as space-time information and covariates to produce modeled estimates, which have been used in study such as the global economic burden of infectious diseases and China’s vaccine economic evaluation.^3,62,76,77^ Besides, that the estimates from the GBD 2019 (0.96, 0.73–1.23 per child) was comparable with that (1.22, 0.53–1.97 per child) from a study that systematically reviewed China’s studies before 2012, considering the declined tread of diarrhea morbidity in China.^31,78^ Moreover, our sensitivity analysis showed that the incidence of RVGE was not the major sensitive parameter that impacted the study conclusion, especially for Rotateq and Rotarix, suggesting it is an acceptable model input. To obtain the ratio of outpatient visits to hospitalization, we have reviewed the studies on rotavirus disease burden and epidemiology in China (Supplementary Materials 2). Among the 4 papers found, a similar ratio of about 10 was reported from regions of different socio-economic levels and tiers of hospitals.^79–81^ We chose the population-based surveillance by Zhang et al., as it reflected the most updated rotavirus epidemiology and healthcare utilization in regions with higher socio-economic (Beijing) and lower socio-economic levels (Gansu) and included the largest number of hospitals to date to ensure the representativeness.^35^ Also, the sensitivity analyses show that the ratio of outpatient visits vs. hospitalization was not the major sensitive parameter for the three RV vaccines, suggesting it is a consistent and acceptable model input.

Similarly, we have conducted a review to collect the cost of illness for rotavirus diarrhea among children under 5 in China (Supplementary Materials 3). Among the 11 studies found, 6 were conducted after 2010, and most of the 6 studies were based on the surveillance in Beijing and Gansu Province.^78–80–82–88^ We obtained the cost data based on the population-based surveillance by Zhang et al., the updated and the only study that covered the regions of different socio-economic levels.^78–80–82–88^ Compared with China’s previous studies, the cost reported by Zhang et al., such as that of the hospitalization, was in the cost range among other studies conducted in rural or urban settings.^78–80–82–88^ It was also comparable with the cost estimates of all-cause diarrhea in China modeled by Baral et.al. in 2015 (Etc., the total cost of outpatient visit: 144.9 vs. 112.1; total cost of hospitalization: 372.3 vs. 453.9).^89^ Moreover, we have made adjustment, extrapolating the local data to the nation. The direct medical costs were adjusted using health care expenditures by region, and direct non-medical costs were adjusted using the consumer price index (CPI).^60,61^ The sensitivity analysis also showed that the costs of health care did not significantly impacting our conclusion.

As the utilities may vary among countries and regions with different socio-economic levels, disease epidemiology, healthcare system, and health service utilization pattern, our study obtained QALY weights from the only study in the developing country, Thailand.^45^ First, the RV epidemiology is similar in China and Thailand, with a relatively high morbidity and low mortality rate.^35,75,90^ For example, the hospitalization rate of RVGE was estimated about 11.3 per 1000 children in Thailand and 14.4/1000 in China, and G9P[8] and G3P[8] are the most dominant RV genotype in Thailand (47.3%) and China (40.4%).^35,75,90^ Second, with the universal health coverage and public hospital system, both countries have promoted health service accessibility, especially primary healthcare, enabling individuals to seek care at nearby health units.^91,92^ Last, surveys reported similar care-seeking behaviors of parents for their children with diarrhea, with a care-seeking rate of about 60% in Thailand and 58–74% in China.^37–39-40–46^ In the absence of local data, QALY weights based on the study in Thailand provided a reasonable estimation.

In addition, the impact of vaccination schedules of different RV vaccines on the immunization delivery cost needed to be clarified. In our study, the cost of routine immunization service per dose was used as the proxy for the three RV vaccines’ immunization delivery costs per dose.^66^ As recommended by WHO, we assumed Rotateq or Rotarix to be delivered in conjunction with the Diphtheria, Tetanus, and Pertussis (DTP) vaccine.^2,9^ For LLR that would be given annually for three doses, the first, second, and third dose could by delivered in conjunction with the DTP vaccine, Japanese encephalitis vaccine, and meningococcal vaccine, respectively, based on China’s national immunization program schedule.^93^ WHO position paper has addressed that simultaneous administration of RV vaccines with these vaccines included in the NIP has not been shown to interfere with the protective immune responses or safety profiles of the respective vaccines.^2,9^ So, the RV vaccination would not require additional visits besides the current routine visits and would not result in a significantly higher immunization delivery cost. Each RV vaccine’s total immunization delivery cost in the NIP depends on the number of doses required.

Consistent with the previous studies in China, which all conducted before 2016, this study suggests the potential cost-effectiveness of the national RV vaccination program, especially using Rotateq or Rotarix.^23–26^ When comparing results within studies, various aspects of factors should be noted, including the input parameters, model settings and threshold for interpreting evaluations.,^29,94,95^ Previous studies have assumed the vaccine prices, one of the most important model parameters, were at a low level (US$1.19 to US$30) compared to the current private market prices.^23–26^ As for the model settings, previous studies assumed zero vaccine coverage in the status quo.^23–26^ However, LLR has been approved for use for twenty years, and the recent investigation after 2018 has reported a first-dose coverage of 32.8% in six provinces, making the private market provision an non-neglectable scenario for comparison.^20^ In addition, our model further considered the significant protection by natural immunity against sequential RV infections, which has been demonstrated by epidemiology studies with birth cohorts and integrated in recent economic evaluations.^28,96,97^ These updates would therefore lead to a relatively conservative cost-effectiveness result in our study compared to previous studies. Researchers have suggested tailored thresholds for economic evaluations to reflect the opportunity cost of health interventions in different settings of countries.^70,71^ For example, Thailand has adopted a threshold of 1.2 times GDP per capita for economic evolution.^98^ In our study, Rotateq or Rotarix through the NIP would be cost-effective at a threshold of GDP per capita but not cost-effective at a more stringent threshold advised by Woods et al.^69^ This suggests that though having significant potential, the inclusion of RV vaccines into the NIP may not reach a clear decision at this time point if other vaccination programs or health interventions, such as sanitation, were to be considered by the policymakers under a limited budget.^70^ More measures are needed to promote the RV vaccination, such as adjusting the vaccine price. The systematic review of RV vaccines showed that the mass vaccination programs tended to be cost-saving or cost-effective in low-and-middle income countries but not in high-income countries, which were mainly due to the high vaccine prices or low to no subsidies in the high-income settings.^94^ Similarly, vaccine prices were also identified as one of the most sensitive parameters in our one-way analyses. The landscape of vaccines in China has highlighted that the contract prices for vaccines in the private market are often higher than prices for comparable vaccines in the US, Europe, and UNICEF.^19^ Furthermore, the RV vaccines are among the costly vaccines ($26- 43/dose) in the Chinese private market, approximately ten times the price in European countries, GAVI Vaccine Alliance-eligible countries, and UNICEF ($0.8–5.1/dose).^19,64,99^ The LLR vaccine is reported to be used mainly in areas with good economic conditions, with extremely limited use in poor areas.^100^ Given the limited budget constraints on investment in vaccination and the relatively low contract prices of vaccines in the NIPs ($0.1–5.7), a reduction in future contract prices for vaccines may be needed to include RV vaccine in the NIP.^19^ The practice is possible, as our scenario analyses demonstrated that the ICERs values for Rotateq and Rotarix would be halved at a 25% reduction in price, approximating the upper bound of the cost-effective threshold range by Woods et al.^69^ The break-even prices, which would help achieve significant public health impact, would be approximately 40% of the current prices. Manufacturers are also likely to benefit through large demands in economies of scale, with a dramatic increase in vaccine coverage from around 20% to over 90%.^101^

In addition to the direct protection of the vaccine on individuals receiving vaccination, the indirect effects, including herd immunity, were indicated to play a role in the population-level impact of vaccination.^102,103^ Recentstudies have attempted to incorporate the herd immunity into the economic evaluations, such as those in the United States, Canada, Japan, and the Netherlands, and the methods have developed from assuming an increase in vaccine effectiveness to generating relational expressions of coverage rate and effectiveness including herd immunity for the static model, or adopting dynamic models.^104,105^ Considering the lack of epidemiological data on rotavirus in China, the relational expressions informed by empirical studies in other countries, including low- and middle-income countries, were adopted in our scenario analyses.^73^ Including herd immunity further improves the cost-effectiveness of Rotateq or Rotarix in comparison with the private market provision with LLR. This is mainly due to the higher efficacies of the two imported vaccines (58.1–78.9%) compared to the domestic LLR (34.9–39%), leading to a larger increment in vaccine effective coverage (VEC) as indicated by the relational expressions.^27,51,54,55^ However, the indirect effect would be less significant when the vaccine coverage reaches over 90% in the mass vaccination scenario instead of at the range between 20–60% in the status quo, making LLR through the NIP less cost-effective compared to the private market provision after including herd immunity in the analyses.

Since 2009, China has been strengthening the surveillance networks for viral diarrhea, including sentinel surveillance and population-based surveillance to provide epidemiological evidence.^106,107^ Regarding the unclear impact of the domestic LLR vaccine, which due to both the lack of RCT and the complicated and delayed schedule for administrating compared with that recommend by the WHO, a novel improved vaccine, the oral human-lamb reassortant trivalent (G2-G3-G4) vaccine has been developed recently.^108^ Moreover, the national expert advisory committee has been established in 2017 to inform evidence-based decision-making and adjustment.^110^ All of these practices would help to promote progress in considering the inclusion of RV vaccines into the NIP. This study highlights the significant public health impact and cost-effectiveness of incorporating RV vaccines in the NIP in China, especially those with better vaccine characteristics (e.g., efficacy) and low prices.

Our study has several limitations. First, the efficacy of domestic LLR vaccine and duration of protection at each dose were estimated from real-world studies on its effectiveness. Second, due to the lack of reports on RV epidemiology in China, the way in which the impact of the herd immunity effect was included in the model was based on the estimation from empirical studies in other countries. The herd immunity may have a relatively different impact in China, as the vaccine coverage in the private market may vary across regions and population groups at different socio-economic levels. Third, some of the model inputs, including the ratio of outpatients visit vs. hospitalization and cost of health care, were based on regional surveillance and surveys, which may hinder the representativeness. Last, the current projection was developed based on the latest data from the ‘pre-COVID-19’ era, while the COVID-19 pandemic may have influenced the transmission and prevention of enteric pathogen infections, as well as the cost-effectiveness of the vaccination. With future updates on local data, studies are needed to better evaluate the impact of vaccination by considering the dynamic transmission of the RV, quantifying its impact on health inequality, and comparing the vaccination programs with other sectors such as water and sanitation investment.

## Conclusion

The national rotavirus vaccination program in China is expected to be cost-effective in comparison with the current provision by private market, reducing substantial disease and economic burden among children under 5 years of age. Reducing vaccine prices, adjusting the current NIP schedule to include rotavirus vaccination, and adopting or developing the vaccine with better characteristics, such as Rotateq or Rotarix, would be the focus of future efforts to promote the introduction of rotavirus vaccine.

## Supplementary Material

Supplementary Material

## Data Availability

The data supporting the findings of this study are available within the article and its supplementary materials.
