# Supplementary material for: Public health impact and cost-effectiveness of rotavirus vaccination in China: Comparison between private market provision and national immunization programs
Source: Hum Vaccin Immunother. 2022 Jul 11;18(7):2090162. doi: 10.1080/21645515.2022.2090162 (PMC10019831; doi:10.1080/21645515.2022.2090162)
Supplement: Supplementary Material [file KHVI_A_2090162_SM4108.docx]

**Supplementary Material 1. Estimation method of including herd immunity effect of rotavirus vaccination**

1. **Weighted Vaccine Coverage**

For the herd immunity sensitivity scenario analysis, we estimated the combined vaccine effect for direct and indirect effects by firstly estimating the weighted vaccine coverage (Table 1).

For Rotateq or Rotarix, the weighted vaccine coverage was estimated using the following formula:

$$C_{w}= \sum_{j} C_{j}R_{j}$$

where *C_w_* is the weighted vaccine coverage, *C_j_* is the coverage for *j* total doses (for Rotateq: j=1, 2, 3; for Rotarix: j=1, 2), and *R_j_* is ratio of the vaccine efficacy of j dose versus the doses of the completed immunization (3 for Rotateq, 2 for Rotarix).

For LLR, due to the lack data by clinic trials, and case-control studies have reported the effectiveness of 1 dose vaccination in the first two years, the weighted vaccine coverage was estimated as:

$$C_{w}= \sum_{j} C_{j}$$

where *C_w_* is the weighted vaccine coverage, *C_j_* is the coverage for *j* total doses (j=1, 2, 3).

The vaccine coverage in the status que, the private market provision with LLR, was estimated from a facility-based survey in 2019, covering over 6,000 children in 10 provinces.^1, 2^ The vaccine coverage in the scenarios of the national immunization programs (NIPs) using the three vaccines, respectively, the DTP vaccine coverage in China was used as a proxy.^3^

**Table 1. Estimation of weighted vaccine coverage (%) of different scenarios**

| **Scenario** | **1 Dose Coverage** | **2 Dose Coverage** | **3 Dose Coverage** | **Weighted Vaccine Coverage** |
| --- | --- | --- | --- | --- |
| The status quo: Private market provision with LLR | 20.26 | 5.51 | 1.77 | 27.54 |
| NIP with Rotateq | 0.13 | 8.39 | 91.05 | 99.29 |
| NIP with Rotarix | 8.39 | 91.05 | / | 98.43 |
| NIP with LLR | 0.13 | 8.39 | 91.05 | 99.57 |

1. **Vaccine Effective Coverage**

The impact of including herd immunity in the model was assessed using the following regression model developed by Wahl et al.^1, 4^ The equations are informed by empirical studies.^5-10^

$${VEC}_{combined}= \left\{ \begin{aligned} C_{w}\times E, &C<14\% \\ \left( 0.962+\ln\left( C_{w} \right)\times0.422 \right)\times(E/0.906), &C \geq14\% \end{aligned} \right.$$

where *VEC_combined_* is the vaccine effective coverage of the combined effect (direct and herd immunity effect), which represents the proportion of disease incident case reduction. *C_w_* is the weighted vaccine coverage. For Rotateq or Rotarix, *E* is the vaccine efficacy from a full course of immunization; For LLR, *E* is the vaccine efficacy of 1 dose. When *C_w_* is <14%, *VEC_combined_* equals the vaccine effective coverage of direct effect only.

**References:**

1. Zhang H, Garcia C, Yu W, Knoll MD, Lai X, Xu T, et al. National and provincial impact and cost-effectiveness of Haemophilus influenzae type b conjugate vaccine in China: a modeling analysis. BMC Medicine 2021;19(1).

2. Lai X, Rong H, Ma X, Hou Z, Li S, Jing R, et al. Willingness to Pay for Seasonal Influenza Vaccination among Children, Chronic Disease Patients, and the Elderly in China: A National Cross-Sectional Survey. Vaccines 2020;8(3):405.

3. Cao L, Wang HQ, Zheng JS, Yuan P, Cao LS, Zhang GM. National Immunization Coverage Survey in China after Integrated more Vaccines into EPI Since 2008. Chin J Vaccines And Immunization.2012; 18(05):419-424+478.

4. Wahl B, O'Brien KL, Greenbaum A, Majumder A, Liu L, Chu Y, et al. Burden of Streptococcus pneumoniae and Haemophilus influenzae type b disease in children in the era of conjugate vaccines: global, regional, and national estimates for 2000-15. Lancet Glob Health 2018;6(7):e744-e757.

5. Fischer Walker CL, Perin J, Aryee MJ, Boschi-Pinto C, Black RE. Diarrhea incidence in low- and middle-income countries in 1990 and 2010: a systematic review. BMC Public Health. 2012;12:220.

6. Yen C, Armero Guardado JA, Alberto P, Rodriguez Araujo DS, Mena C, Cuellar E, et al. Decline in rotavirus hospitalizations and health care visits for childhood diarrhea following rotavirus vaccination in El Salvador. Pediatr Infect Dis J. 2011;30(1 Suppl):S6-S10.

7. Yen C, Tate JE, Wenk JD, Harris JM, 2nd, Parashar UD. Diarrhea-associated hospitalizations among US children over 2 rotavirus seasons after vaccine introduction. Pediatrics. 2011;127(1):e9-e15.

8. Cortese MM, Tate JE, Simonsen L, Edelman L, Parashar UD. Reduction in gastroenteritis in United States children and correlation with early rotavirus vaccine uptake from national medical claims databases. Pediatr Infect Dis J. 2010;29(6):489-94.

9. Payne DC, Staat MA, Edwards KM, Szilagyi PG, Weinberg GA, Hall CB, et al. Direct and indirect effects of rotavirus vaccination upon childhood hospitalizations in 3 US Counties, 2006-2009. Clin Infect Dis. 2011;53(3):245-53.

10. Panozzo CA, Becker-Dreps S, Pate V, Weber DJ, Jonsson Funk M, Sturmer T, et al. Direct, indirect, total, and overall effectiveness of the rotavirus vaccines for the prevention of gastroenteritis hospitalizations in privately insured US children, 2007-2010. Am J Epidemiol. 2014;179(7):895-909.

**Supplementary Material 2. Review on Rotavirus Disease Burden and Epidemiology among Children Under 5 in China to Obtain the Ratio of Outpatient visit vs. Hospitalization**

To obtain the ratio, we have conducted a review on rotavirus disease burden and epidemiology among children under 5 in China. We searched both Chinese and English papers in PubMed, Embase, CNKI (知网), and Wanfang (万方) databases, and sought papers that reported rotavirus diarrhea incidence by settings (etc. hospitalization, inpatient) and adopted a surveillance design. Four studies met the criteria. The results are shown below in Table 1.

Among the 4 papers in China, a similar ratio was reported from regions of different socioeconomic levels and tiers of hospitals. The two population-based surveillances (Fang et al., 2005; Fang et al., 2007) in the rural area (Lulong County), where the village doctor visited families to collect data every month, reported the ratios (11.8 and 14.7) of the number of community vs. hospitalization cases. Considering a care-seeking rate of RV diarrhea of about 75%, the ratio of outpatient visits vs. hospitalization could be estimated to be about 7.9 to 10.2. Besides, the two surveillances based on secondary and tertiary hospitals, covering regions of higher socio-economic level (Beijing, Hangzhou) or lower socio-economic level (Gansu), reported similar ratios (9.6 and 10.3) of outpatients visit vs. hospitalization. So, based on the available evidence, setting the ratio of outpatients visit vs. hospitalization as 10 may be reasonable and consistent across regions and settings.

Table 1 Surveillance of rotavirus disease burden and epidemiology among U5 children in China

| Reference | Study Time | Province | Study Place | Study Design | Involved hospital level | Incidence | | | Ratio (setting) |
| --- | --- | --- | --- | --- | --- | --- | --- | --- | --- |
|  |  |  |  |  |  | Community | Outpatient | Inpatient |  |
| Fang et al., 2005 | 1999.7-2003.6 | Hebei | Lulong county | Population- and hospital-based Surveillance | Primary | 130/1000 | / | 11/1000 | 11.8 (Community vs. hospitalization) |
| Fang et al., 2007 | 1999.7-2002.6 | Hebei | Lulong county | Population- and hospital-based Surveillance | Primary | 131/1000 | / | 8.9/1000 | 14.7 (Community vs. hospitalization) |
| Lou et al., 2011 | 2007.1-2008.12 | Zhejiang | Hangzhou | Hospital-based Surveillance | Secondary and tertiary | / | 20.1/1000 | 2.1/1000 | 9.6 (Outpatient vs. hospitalization) |
| Zhang et al., 2015 | 2012.7-2013.6 | Beijing, Gansu | Beijing, Gansu | Hospital-based Surveillance | Secondary and tertiary | / | 14.9/1000 | 1.44/1000 | 10.3 (Outpatient vs. hospitalization) |

**Reference:**

[1] Fang Z, Zhang L, Tang J, Zhang Q, Hu H, Xie H, et al. Study on Rotavirus Diarrhea among Children in Lulong County, Hebei Province, China. Chinese Journal of Virology, 2005(01):21-26. (in Chinese)

[2] Tang J, Fang Z, Hu H, Ye Q, Gao F, Liu C, et al. Study on disease burden and character of rotavirus diarrhea among children in Lulong county of China. Chinese Journal of Child Health care, 2007(02):179-180. (in Chinese)

[3] Lou J, Xu X, Wu Y, Tao R, Tong M. Epidemiology and burden of rotavirus infection among children in Hangzhou, China. Journal of Clinical Virology 2011;50(1):84-87.

[4] Zhang J, Liu H, Jia L, Payne DC, Hall AJ, Xu Z, et al. Active, Population-based Surveillance for Rotavirus Gastroenteritis in Chinese Children: Beijing Municipality and Gansu Province, China. Pediatric Infectious Disease Journal. 2015;34(1):40-46.

**Supplementary Material 3. Review on the Cost of Illness for Rotavirus Diarrhea among Children Under 5 in China to Obtain the Cost of Health Care**

Similarly, we reviewed the cost of illness for rotavirus diarrhea among children under 5 in China. We searched both Chinese and English papers in PubMed, Embase, CNKI (知网), and Wanfang (万方) databases, and collected data on direct medical, direct non-medical, and indirect costs of a rotavirus diarrhea episode at health care facilities and in different settings (homecare, visit ,or hospitalization). The results were presented below in Table 2 in the currency at the time of study and in 2019 USD$ for comparison.

Among the 11 studies found, only 6 were conducted after 2010. Besides, most of the 6 studies were based on the surveillance in Beijing and Gansu Province. Considering the available evidence, we obtained the cost data based on the population-based surveillance (Zhang et al., 2015). It is the updated and the only study covering regions of different socio-economic levels with representativeness, at least at the provincial level. Compared with other studies in China, the cost data reported by Zhang et al., such as that of the hospitalization, was in the cost range among other studies in rural or urban settings. In addition, the empirical cost of rotavirus diarrhea reported by Zhang et al. was also comparable with the cost estimates of all-cause diarrhea in China modelled by Baral et.al. in 2015 (Etc., the total cost of outpatient visit: 144.9 vs. 112.1; total cost of hospitalization: 372.3 vs. 453.9).

Table 1 Cost of illness for rotavirus diarrhea among U5 children in China

| Reference | Study Time | Province | Study Place | Currency, year | Outpatient | | | Inpatient | | | Outpatient (adjusted to 2019, USD) | | | Inpatient (adjusted to 2019, USD) | | |
| --- | --- | --- | --- | --- | --- | --- | --- | --- | --- | --- | --- | --- | --- | --- | --- | --- |
|  |  |  |  |  | Direct medical cost | Direct non-medical cost | Indirect cost | Direct medical cost | Direct non-medical cost | Indirect cost | Direct medical cost | Direct non-medical cost | Indirect cost | Direct medical cost | Direct non-medical cost | Indirect cost |
| Wu et al., 2003 | 2001.9-2002.3 | Jiangsu | Children's Hospital of Suzhou | 2002, RMB | / | / | / | 1686.0 | 102.0 | 306.0 | / | / | / | 400.4 | 24.2 | 72.7 |
| Zhang et al., 2005 | 2005.1-2005.3 | Hebei | Lulong County Hospital | 2005, RMB | 89.0 | 22.0 | 9.0 | 676.0 | 166.0 | 83.0 | 19.8 | 4.9 | 2.0 | 150.3 | 36.9 | 18.5 |
|  | 2005.1-2005.3 | Jilin | Children's Hospital of Changchun | 2005, RMB | 212.0 | 37.0 | 18.0 | 1667.0 | 253 .0 | 229.0 | 47.1 | 8.2 | 4.0 | 370.6 | 56.2 | 50.9 |
| Tang et al., 2007 | 1999.7-2004.6 | Hebei | Lulong County Hospital, Maternal and child health hospital of Lulong County | 2004, RMB | / | / | / | 676 | 166.0 | 83.4 | / | / | / | 153.0 | 37.6 | 18.9 |
| Xiong et al., 2010 | 2009.1-2009.12 | Guangdong | Shenzhen Longgang District | 2009, RMB | 218.0 | / | / | 3201.0 | / | / | 43.3 | / | / | 635.6 | / | / |
| Jin et al., 2011 | 2006.10-2007.12 | Beijing, Shanghai, Jiangsu, Fujian, Guangdong | Capital Institute for Pediatrics, Pediatric Hospital of Fudan University, Children Hospital of Suzhou, Fujian Provincial Hospital, Guangzhou Children’s Hospital | 2007, USD | 14.0 | 14.9 | 20.9 | 3402.7 | 265.1 | 700.6 | 21.6 | 22.9 | 32.3 | 710.5 | 55.4 | 146.3 |
| Liu et al., 2013 | 2012.7-2013.2 | Beijing, Gansu | 5 sentimental hospitals | 2013, RMB | 354.0 | 87.5 | 343.6 | 1150.5 | 138.4 | 687.1 | 61.3 | 15.1 | 59.5 | 199.1 | 23.9 | 118.9 |
| Liu et al., 2014 | 2012.8-2013.7 | Gansu | 2 sentimental hospitals | 2013, RMB | 375.4 | 47.9 | 270.5 | 2053.9 | 180.1 | 721.2 | 65.0 | 8.3 | 46.8 | 355.5 | 31.2 | 124.8 |
| Jia et al., 2015 | 2012.7-2013.7 | Beijing | 3 children's hospitals | 2013, RMB | 150.4 | / | 304.4 | / | / | / | 26.0 | / | 52.7 | / | / | / |
| Zhang et al., 2015 | 2012.7-2013.6 | Beijing, Gansu | 6 setimental hospitals (Beijing Children’s Hospital, Peking University First Hospital, Capital Pediatric Research Institute, Tong Zhou District Women, Children’s Medical Center, Lanzhou University Hospital, Liangzhou District Hospital of Wuwei city) | 2013, USD | 58.0 | 18.8 | 68.1 | 204.5 | 39.6 | 128.2 | 61.1 | 19.8 | 71.7 | 215.4 | 41.7 | 135.0 |
| Cui et al., 2016 Li et al., 2019 | 2014.10-2015.9 | Jiangsu | Children's Hospital affiliated to Nanjing Medical University | 2015, RMB | 241.7 | 228.1 | 500.6 | 3019.5 | 1144.8 | 3461.8 | 40.5 | 38.2 | 83.9 | 505.8 | 191.7 | 579.8 |

**Reference:**

[1] Wu S, Wang B, Jin H, Wang N. Surveillance and estimates of disease burden on group A rotavirus gastroenteritis. Journal of Southeast University (Medical Science Edition), 2003(02):80-83. (in Chinese)

[2] Zhang L. Rotavirus Surveillance and Molecular Epidemiology among children with acute diarrhea in six regions of China (Doctoral Thesis). Chinese Center for Disease Control and Prevention, Beijing, China. 2005. (in Chinese)

[3] Tang J, Fang Z, Hu H, Ye Q, Gao F, Liu C, et al. Study on disease burden and character of rotavirus diarrhea among children in Lulong county of China. Chinese Journal of Child Health care, 2007(02):179-180. (in Chinese)

[4] Xiong L, Lin S, Li H. STUDY ON THE INCIDENCE AND ECONOMIC BURDEN OF ROTAVIRUS DIARRHEA IN LONGGANG DISTRICT OF SHENZHEN. Modern Preventive Medicine, 2010;37(12):2332-2333. (in Chinese)

[5] Jin H, Wang B, Fang Z, Duan Z, Gao Q, Liu N, et al. Hospital-based study of the economic burden associated with rotavirus diarrhea in eastern China. Vaccine. 2011;29(44):7801-6.

[6] Liu H, Study on disease burden of rotavirus diarrhea among children under five years old in Beijing and Gansu (Master Thesis). Chinese Center for Disease Control and Prevention, Beijing, China. 2013. (in Chinese)

[7] Liu H, Liu X, Liu D, Zhang J, Meng L. Analysis on Economic Burden Evaluation and Epidemiology of Rotavirus Diarrhea among Children under Five Years Old. Chinese Primary Health Care. 2014;28(06):90-92. (in Chinese)

[8] Jia L, Liu X, Li H, Liang Y, Gao Z, Wang Q. Study on the Economic Burden of Rotavirus Diarrhea among Children under 5 Years Old in Beijing. Chinese Journal of Vaccines and Immunization. 2015;21(05):543-546+551. (in Chinese)

[9] Zhang J, Liu H, Jia L, Payne DC, Hall AJ, Xu Z, et al. Active, Population-based Surveillance for Rotavirus Gastroenteritis in Chinese Children: Beijing Municipality and Gansu Province, China. Pediatric Infectious Disease Journal. 2015;34(1):40-46.

[10] Cui P. Study on disease burden of rotavirus diarrhea and epidemiologic characteristic of intussusception among children in China (Master Thesis). Chinese Center for Disease Control and Prevention, Beijing, China. 2016. (in Chinese)

[11] Li J, Cui P, Tao Y, Jin Y, Liu N. Economic burden of rotavirus diarrhea in children under 5 years old in Nanjing. International Journal of Virology. 2019(04):229-232. (in Chinese)

[12] Baral R, Nonvignon J, Debellut F, Agyemang SA, Clark A, Pecenka C. Cost of illness for childhood diarrhea in low- and middle-income countries: a systematic review of evidence and modelled estimates. BMC Public Health 2020;20(1).
